# Supplementary material for: Many-objective African vulture optimization algorithm: A novel approach for many-objective problems
Source: PLoS One. 2023 May 17;18(5):e0284110. doi: 10.1371/journal.pone.0284110 (PMC10191289; doi:10.1371/journal.pone.0284110)
Supplement: Attachment — Submitted filename: Response to Reviewers v5.docx [file pone.0284110.s001.docx]

**Response to Reviewers**

Manuscript Number: **PONE-D-22-28481**

The title **“Many-objective African vulture optimization algorithm for solving the series-parallel system problem of real-life constrained engineering”**

The authors would like to thank the Editors and the Reviewers for their valuable and constructive comments and suggestions, which guided us toward a substantial revision of the paper.

**We suggest changing the paper title to “Many-Objective African Vulture Optimization Algorithm: A Novel Approach for Many-Objective Problems” to be more expressive of the paper content after additions and modifications.**

All the reviewer comments and suggestions have been considered and covered in the new version of the paper. Note that the new parts are highlighted in yellow color in the revised manuscript, but in answer to the reviewers' letter, the structure is as follows:

- Phrases in light blue cells: Reviewer remarks and comments

- Phrases in white cells: Author response and explanation to the reviewer remarks and comments.

**Reviewer (1)**

This paper presents a many-objective African vulture optimization algorithm for solving the series-parallel system problem of real-life constrained engineering. Before considering it for publication, the following comments should be revised carefully:
1- The abstract should be improved including the novelties and software used in the presented work.

Thank you for your comment. We appreciate your suggestion to improve the abstract of the paper. We have already revised the abstract to include a more detailed description of the novelties of our proposed algorithm, as well as the software used in the presented work. The revised abstract accurately reflects the content of the paper and effectively conveys the contributions of our work. Thank you again for your constructive comments.

2- English typos and grammatical errors must be revised.

Thank you for your comment. We have reviewed and corrected any English typos and grammatical errors in the paper. We appreciate you bringing this to our attention and helping us improve the quality of our work.

3- Some parameters are not defined.

We have carefully reviewed the paper and added a section that defines all of the parameters used in our proposed algorithm. In addition, we have included Table 1 in the paper, which provides a summary of all the variables and their definitions. We have highlighted these changes in Section 2 and Section 3 of the paper to ensure that they are easily identifiable. Thank you for your constructive comments and for helping us improve the quality of our work.

4- Please double-check all the equations.

We have carefully double-checked the equations and made sure that they are properly formatted and easy to read. We appreciate you bringing this to our attention and helping us improve the quality of our work.

5- The parameters of the used algorithms should be provided in a table.

Certainly, we appreciate the reviewer's suggestion. In response to this comment, we have added a new table in our manuscript, Table 1, that provides the parameters used in the algorithm.

6- Different population and iteration sizes should be implemented to show the accuracy of the presented technique.

Thank you for your feedback. We appreciate your suggestion regarding the use of different population and iteration sizes to demonstrate the accuracy of the proposed technique.

We have used multiple iteration sizes in the implementation of the engineering problems to demonstrate the effectiveness of our algorithm. However, due to the limitations of paper size, we could not include all the results in the manuscript.

Additionally, the choice of population size and iteration size can be influenced by several factors, including the number of objectives, the distribution of the reference directions, and the available computational resources. Therefore, it is not possible to define a single optimal population and iteration size for all scenarios.

Nonetheless, we have made our code available to the readers to facilitate the replication and evaluation of our proposed algorithm using different population and iteration sizes. We believe that the results obtained using different population and iteration sizes will further validate the effectiveness of our algorithm.

Once again, thank you for your valuable feedback, which helped us to improve the quality of our manuscript.

7- The resolution of some Figs should be improved.

Thank you for bringing the resolution of some figures to our attention. We have taken steps to improve the resolution of those figures and ensure that they are clear and easy to read.

8- A recent optimization techniques should be implemented for comparison like YUKI algorithm, ….. .

We are pleased to inform you that we have added the comparative results of another many-objective optimization algorithm, AGEMOEA, to the paper. However, we would like to clarify that the YUKI algorithm is a new metaheuristic algorithm specifically designed for solving identification problems and is not a many-objective optimization algorithm like our proposed MAAVOA algorithm.

To ensure a fair comparison, we compare MAAVOA with other many-objective optimization algorithms, such as NSGAIII, U-NSGA-II, MOEA/D, CTAEA, and AGE-MOEA, which are commonly used in such comparisons.

9- The introduction should be improved including more references based on the most used optimization techniques in engineering applications such as: damage identification, characterization, …. . Composite Structures 6 October 2022, 116272_Mechanics of Materials Volume 166, March 2022, 104200 _ Met. Mater. Int. 28, 370–384 (2022)_ Mechanics Volume 118, April 2022, 103213_Journal of Computational Science Volume 55, October 2021, 101451_Expert Systems with Applications Volume 186, 30 December 2021, 115669_Theoretical and Applied Fracture Mechanics Volume 107, June 2020, 102554_ Engineering Fracture Mechanics Volume 205, January 2019, Pages 285-300.

Thank you for your suggestion. We have included additional comparative results and discussions in our revised manuscript to provide a more rigorous and accurate validation of our proposed algorithm. Moreover, we have included a new engineering application in Section 5.2 to demonstrate the practicality of our algorithm. While we acknowledge that real experimental validation is important, due to practical limitations, it is not feasible to conduct extensive real-world experiments in many-objective optimization. Nonetheless, we believe that the extensive comparative results and the new application provide strong evidence for the effectiveness of the proposed algorithm.

10- The conclusion should be improved and show the limitation of the presented technique.

Thank you for your suggestion. We have revised the conclusion section to include a summary of the experimental results and highlight the superiority of our proposed algorithm. we have presented the relevant data and analysis in the Results section. We hope that these revisions address your concerns and improve the clarity of our conclusions.

**Reviewer (2)**

1. The contribution is not stated clearly.

Thank you for your comment. We have added a section in the introduction highlighting our work's main contributions. We hope that this will provide a clearer understanding of the novelty and significance of our proposed algorithm. Please let us know if you have any further suggestions or comments.

2. The choice of parameters used in the algorithm is not well justified.

Thank you for your feedback. We have revised the manuscript to include a table (Table 1) that outlines the parameters used in the proposed algorithm. We believe that this will provide more transparency and clarity on the choices made for the parameters. We hope this addresses your concern and appreciate your valuable feedback.

3. A deep and detailed comparison with other methods is mandatory.

We are delighted to inform you that we have incorporated the comparative results of another many-objective optimization algorithm, AGEMOEA, in our paper. Moreover, we have included a new engineering application in Section 5.2. We hope that these additions contribute significantly to the quality of our work and address the concerns raised by the reviewers.

4. The authors claim that their method is faster and more efficient, but this is not rigorously demonstrated since it is applied just for a particular case.

The proposed algorithm is demonstrated and compared to five well-known algorithms (one new algorithm is add to the revised version) in performing 7 standard optimization problems (DTLZ functions 1-7) in 5 different values of objectives (3,5,8,10,15) and is applied and compared in case of two practical engineering applications (one new engineering application is add to the revised version). We hope the additional experiments to the revised version are enough to ensure our claim.

5. What do you mean by experimental validation? Where the data exactly comes from, what is their reliability and accuracy for which model? Please address this important point seriously. Authors must cite the following papers; Overall the quality of this paper is very good.
I recommend this paper. Authors must cite the following papers:

- Singh, N., Hamid, Y., Juneja, S., Srivastava, G., Dhiman, G., Gadekallu, T. R., & Shah, M. A. (2023). Load balancing and service discovery using Docker Swarm for microservice-based big data applications. Journal of Cloud` Computing, 12(1), 1-9.
- Nayak, J., Swapnarekha, H., Naik, B., Dhiman, G., & Vimal, S. (2022). 25 Years of Particle Swarm Optimization: Flourishing Voyage of Two Decades. Archives of Computational Methods in Engineering, 1-63.
- Zhen, S., Surender, R., Dhiman, G., Rani, K. R., Ashifa, K. M., & Reegu, F. A. (2022). Intelligent-based ensemble deep learning model for security improvement in real-time wireless communication. Optik, 271, 170123.
- Singh, Shailendra Pratap, Wattana Viriyasitavat, Sapna Juneja, Hani Alshahrani, Asadullah Shaikh, Gaurav Dhiman, Aman Singh, and Amandeep Kaur. "Dual adaption based evolutionary algorithm for optimized the smart healthcare communication service of the Internet of Things in smart city." Physical Communication 55 (2022): 101893.
- Rani, S., Babbar, H., Srivastava, G., Gadekallu, T. R., & Dhiman, G. (2022). Security Framework for Internet of Things based Software Defined Networks using Blockchain. IEEE Internet of Things Journal.
- Singamaneni, K. K., Nauman, A., Juneja, S., Dhiman, G., Viriyasitavat, W., Hamid, Y., & Anajemba, J. H. (2022). An Efficient Hybrid QHCP-ABE Model to Improve Cloud Data Integrity and Confidentiality. Electronics, 11(21), 3510.
- Shukla, Surendra Kumar, Bhaskar Pant, Wattana Viriyasitavat, Devvret Verma, Sandeep Kautish, Gaurav Dhiman, Amandeep Kaur, Kannan Srihari, and Sachi Nandan Mohanty. "An integration of autonomic computing with multicore systems for performance optimization in Industrial Internet of Things." IET Communications (2022).
- Singh, S. P., Dhiman, G., Viriyasitavat, W., & Kautish, S. (2022). A Novel Multi-Objective Optimization Based Evolutionary Algorithm for Optimize the Services of Internet of Everything. IEEE Access, 10, 106798-106811.
- Alrashed, Fahad Abdulaziz, Abdulrahman M. Alsubiheen, Hessah Alshammari, Sarah Ismail Mazi, Sara Abou Al-Saud, Samha Alayoubi, Shaji John Kachanathu et al. "Stress, Anxiety, and Depression in Pre-Clinical Medical Students: Prevalence and Association with Sleep Disorders." Sustainability 14, no. 18 (2022): 11320.
- Singamaneni, K. K., Dhiman, G., Juneja, S., Muhammad, G., AlQahtani, S. A., & Zaki, J. (2022). A novel QKD approach to enhance IIOT privacy and computational knacks. Sensors, 22(18), 6741.
- Ahmad, F., Shahid, M., Alam, M., Ashraf, Z., Sajid, M., Kotecha, K., & Dhiman, G. (2022). Levelized Multiple Workflow Allocation Strategy under Precedence Constraints with Task Merging in IaaS Cloud Environment. IEEE Access, 10, 92809-92827.
- Kumar, R., & Dhiman, G. (2021). A Comparative Study of Fuzzy Optimization through Fuzzy Number. International Journal of Modern Research, 1, 1-14.
- Chatterjee, I. (2021). Artificial Intelligence and Patentability: Review and Discussions. International Journal of Modern Research, 1, 15-21.
- Vaishnav, P.K., Sharma, S., & Sharma, P. (2021). Analytical Review Analysis for Screening COVID-19. International Journal of Modern Research, 1, 22-29.
- Gupta, V. K., Shukla, S. K., & Rawat, R. S. (2022). Crime tracking system and people’s safety in India using machine learning approaches. International Journal of Modern Research, 2(1), 1-7.
- Sharma, T., Nair, R., & Gomathi, S. (2022). Breast Cancer Image Classification using Transfer Learning and Convolutional Neural Network. International Journal of Modern Research, 2(1), 8-16.
- Shukla, S. K., Gupta, V. K., Joshi, K., Gupta, A., & Singh, M. K. (2022). Self-aware Execution Environment Model (SAE2) for the Performance Improvement of Multicore Systems. International Journal of Modern Research, 2(1), 17-27

We greatly appreciate your valuable feedback and suggestion. In response, we have taken action by citing 4 references of the suggested refrences, which are now included in the revised version as ref. numbers 3-6.

6. The authors should provide other applications of the proposed algorithm.To demonstrate the effectiveness of the proposed algorithm a real experimental validation is mandatory for a rigorous and accurate comparison and validation. Please discuss the performance of the technique for real-time applications.

Thank you for your suggestion. We have included additional comparative results and discussions in our revised manuscript to provide a more rigorous and accurate validation of our proposed algorithm. Moreover, we have included a new engineering application in Section 5.2 to demonstrate the practicality of our algorithm. While we acknowledge that real experimental validation is important, due to practical limitations, it is not feasible to conduct extensive real-world experiments in many-objective optimization. Nonetheless, we believe that the extensive comparative results and the new application provide strong evidence for the effectiveness of the proposed algorithm.

7. Specify the limitations and drawbacks of the proposed method. The conclusion must be rewritten.

Thank you for your suggestion. We have revised the conclusion section to include a summary of the experimental results and highlight the superiority of our proposed algorithm. we have also presented the limitations and drawbacks of the proposed model. We hope that these revisions address your concerns and improve the clarity of our conclusions.

**Reviewer (3)**

Suggest the authors highlight the main contributions of their work.

Thank you for your suggestion. We have added a section in the introduction that highlights the main contributions of our work. We hope that this will provide a clearer understanding of the novelty and significance of our proposed algorithm. Please let us know if you have any further suggestions or comments.

A clear statement about contributions should be given in the Introduction section.

Thank you for your comment. We appreciate your suggestion and have added a clear statement about the contributions of our work to the introduction section. We believe that this change makes the main contributions of the paper more evident to the reader. If you have any further feedback on the introduction or any other part of the paper, please let us know. We would be happy to make any further improvements based on your feedback.

Suggest the authors have a solid statement about the proposed method by providing a motivational example.

The revised version includes many clarifications about our work and additional experiments and applications. We hope that it will cover your valuable suggestion.

Suggest the authors add more experimental results.

Thank you for the valuable feedback. We are pleased to inform you that we have updated our paper by adding a comparison with another many-objective optimization algorithm, AGEMOEA, in results Section, and included a new engineering application in Section 5.2. We have also highlighted the main contributions of our work in the Introduction section to better convey the novelty of our approach. We believe these additions significantly improve the quality of our work and address the concerns raised by the reviewers.

The abstract needs to update.

We appreciate your feedback and have revised the abstract of our paper to improve its clarity and reflect the updates we made to the manuscript. We believe that the revised abstract now better highlights the main contributions and outcomes of our work.

The experimental results in this paper are also somewhat weak so need to verify your proposed method in the present form.

We appreciate the reviewer's concern about the experimental results, and we would like to confirm that we have conducted extensive experiments to verify the effectiveness of the proposed algorithm. Specifically, we have compared our algorithm with several state-of-the-art algorithms on many-objective benchmark problems, and we have also included a new engineering application in Section 5.2 to demonstrate the practicality of our algorithm. These results provide strong evidence for the effectiveness of the proposed algorithm. However, we acknowledge that there is always room for improvement, and we will consider incorporating further experiments and analysis in future work.

The contribution of the paper can be neatly elaborated by including the novelty of the paper and the major difference from available models.

Thank you for this comment. We have added a section in the introduction highlighting our work's main contributions. We hope that this will provide a clearer understanding of the novelty and significance of our proposed algorithm. Please let us know if you have any further suggestions or comments.

The author's research is very meaningful, and I hope you will explain the experiment further.

Thanks a lot for your encouraging comment. We hope that the revised version and the additional results and discussions clear our work.

The technical details make it much more intelligible, so please provide some strong technical details in the main methodology.

We add more results and discussions. In addition, a link to the code was added to make it easy for the reader to run and ensure our proposal. The code is enhanced to be more readable.

The researchers are recommended to revise the result section because the comparative results with the existing approaches are not explained briefly

Thanks a lot for your comment. The revised version is enhanced to more results and more discussions. We hope that you will be satisfied with these modifications and additions.

The conclusion should indicate the experimental evaluation's implications and include some obtained values to point out the superiority.

Thank you for your suggestion. We have revised the conclusion section to include a summary of the experimental results and highlight the superiority of our proposed algorithm. we have presented the relevant data and analysis in the Results section. We hope that these revisions address your concerns and improve the clarity of our conclusions.
